# Supplementary material for: Perioperative Analgesia and Patients’ Satisfaction in Spinal Anesthesia for Cesarean Section: Fentanyl Versus Morphine
Source: J Clin Med. 2023 Oct 3;12(19):6346. doi: 10.3390/jcm12196346 (PMC10573232; doi:10.3390/jcm12196346)
Supplement: Supplementary file 1 [file jcm-12-06346-s001.zip › jcm-2609745-supplementary.pdf]

Table S1. Pain in patients who required rescue analgesia.

Morphine group						
**Age in years**	**Smoker**			**Satisfaction**	**AUPS72hR**	**AUPS 72hM**
28	–	*fentanyl 50 mcg intraoperatively*		5	-29	-28
29	–	*fentanyl 50 mcg intraoperatively*		5	0	114
29	Yes	*50 mg tramadol one dose in day 0*		5	0	191
Fentanyl group						
**Age in years**	**Smoker**			**Satisfaction**	**AUPS72hR**	**AUPS72hM**
21	–	*50 mg tramadol*	*two doses*	3	146	350
22	–	*50 mg tramadol*	*two doses*	4	193	297
27	–	*50 mg tramadol*	*two doses*	3	205	296
30	–	*50 mg tramadol*	*three doses*	2	296	466
33	–	*50 mg tramadol*	*two doses*	3	86	372
35	–	*50 mg tramadol*	*one dose*	4	86	188
36	–	*50 mg tramadol*	*one dose*	4	21	301
40	–	*50 mg tramadol*	*one dose*	4	16	196
42	–	*50 mg tramadol*	*two doses*	3	211	388
50	–	*50 mg tramadol*	*one dose*	3	86	171

Table S2. Analgesia-related adverse effects in patients who required rescue medication for pain.

Morphine group									
**Age in years**	**Smoker**			**Satisf**	**Pruritus**	**Nausea**	**Vomiting**	**Const. dizziness**	**Effective treat.**
28	–	*fentanyl 50 mcg intraoperatively*		5	Yes	–	–	–	Yes
29	–	*fentanyl 50 mcg intraoperatively*		5	2	Yes	Yes	Yes	Yes
29	Yes	*50 mg tramadol one dose in day 0*		5	Yes	–	–	Yes	Yes
Fentanyl group									
**Age in years**	**Smoker**			**Satisf**	**Pruritus**	**Nausea**	**Vomiting**	**Const. dizziness**	**Effective treat.**
21	–	*50 mg tramadol*	*two doses*	3	Yes	Yes	–	–	–
22	–	*50 mg tramadol*	*two doses*	4	–	–	–	–	–
27	–	*50 mg tramadol*	*two doses*	3	Yes	–	–	Yes	–
30	–	*50 mg tramadol*	*three doses*	2	–	–	–	–	–
33	–	*50 mg tramadol*	*two doses*	3	Yes	–	–	Yes	–
35	–	*50 mg tramadol*	*one dose*	4	Yes	–	–	–	Yes
36	–	*50 mg tramadol*	*one dose*	4	–	–	–	Yes	–
40	–	*50 mg tramadol*	*one dose*	4	–	–	–	–	–
42	–	*50 mg tramadol*	*two doses*	3	Yes	Yes	–	–	–
50	–	*50 mg tramadol*	*one dose*	3	–	–	–	–	–
